# “I was worried if I don’t have a broken leg they might not take it seriously”: Experiences of men accessing ambulance services for mental health and/or alcohol and other drug problems

**DOI:** 10.1111/hex.12886

**Published:** 2019-04-04

**Authors:** Nyssa Ferguson, Michael Savic, Terence V. McCann, Kate Emond, Emma Sandral, Karen Smith, Louise Roberts, Emma Bosley, Dan I. Lubman

**Affiliations:** ^1^ Turning Point Eastern Health Melbourne Victoria Australia; ^2^ Eastern Health Clinical School Monash University Melbourne Victoria Australia; ^3^ Department of Nursing and Midwifery, College of Health and Biomedicine Victoria University Melbourne Victoria Australia; ^4^ Department of Rural Nursing and Midwifery, College of Health, Science and Engineering La Trobe University Bendigo Victoria Australia; ^5^ Department of Epidemiology and Preventative Medicine Monash University Clayton Victoria Australia; ^6^ Ambulance Victoria Melbourne Victoria Australia; ^7^ Department of Community Emergency Health and Paramedic Practice Monash University Clayton Victoria Australia; ^8^ Department of Paramedics Flinders University Adelaide South Australia Australia; ^9^ Queensland Ambulance Service Brisbane Queensland Australia; ^10^ School of Clinical Sciences Queensland University of Technology Brisbane Queensland Australia

**Keywords:** alcohol and other drugs, ambulance services, mental health, paramedics, qualitative research, stigma

## Abstract

**Background:**

A large proportion of ambulance callouts are for men with mental health and/or alcohol and other drug (AOD) problems, but little is known about their experiences of care. This study aimed to describe men's experiences of ambulance care for mental health and/or AOD problems, and factors that influence their care.

**Methods:**

Interviews were undertaken with 30 men who used an ambulance service for mental health and/or AOD problems in Australia. Interviews were analysed using the Framework approach to thematic analysis.

**Results:**

Three interconnected themes were abstracted from the data: (a) professionalism and compassion, (b) communication and (c) handover to emergency department staff. Positive experiences often involved paramedics communicating effectively and conveying compassion throughout the episode of care. Conversely, negative experiences often involved a perceived lack of professionalism, and poor communication, especially at handover to emergency department staff.

**Conclusion:**

Increased training and organizational measures may be needed to enhance paramedics' communication when providing care to men with mental health and/or AOD problems.

## 1 INTRODUCTION

Men frequently experience mental health and/or alcohol and other drug (AOD) concerns, yet are often reluctant to seek professional help. In 2014‐2015, around 1.5 million Australian men had a self‐reported mental or behavioural condition,^1^ and suicide was the leading cause of death in men aged between 15 and 44 years.^2^ Furthermore, men are more likely than women to experience alcohol and other drug problems.^3^

Men access general practitioners and mental health services significantly less than women do.^4^, ^5^ Common barriers to men's help‐seeking for mental health and/or AOD problems include masculine norms around stoicism, stigma, embarrassment, poor recognition and/or communication of symptoms or concerns.^5^, ^6^, ^7^ When men do present for help, it is most often in the context of an acute physical illness or crisis, rather than help‐seeking for mental health issues as the primary complaint.^8^

Ambulance services are a key front‐line emergency service, attending over 1.3 million emergency incidents annually, of which many are likely to include men with mental health and/or AOD issues.^9^ Indeed, paramedics report that attending mental health and/or AOD presentations comprises a significant proportion of their workload.^10^, ^11^ Australian studies have shown that between 10% and 20% of ambulance presentations are related to mental health and/or AOD concerns.^12^, ^13^ Similar to stigmatizing attitudes documented in the general population and amongst some health professional groups,^14^, ^15^, ^16^ research indicates that some paramedics display negative attitudes towards people who present with mental health and/or AOD problems.^10^, ^11^, ^17^ Such negative attitudes may act as a barrier to help‐seeking and recovery in this population.^18^

Despite this, little is known about the experiences and perceptions of paramedic care amongst people who present with mental health and/or AOD concerns. Studies relating to patient satisfaction have been criticized, as they are often not underpinned by any theoretical or conceptual development and may not accurately reflect patient experiences.^19^, ^20^ The relatively small body of literature around patient experiences of ambulance services is focused on factors associated with patient satisfaction irrespective of the presenting concerns^19^ or key outcomes valued by patients broadly.^21^

As paramedics are often the first health‐care professionals encountered by men experiencing mental health and/or AOD problems, it is important to better understand men's experiences of care. This is because experiences of paramedic care have the potential to act as either a barrier or enabler to accessing future care in the community, and therefore affect their recovery and future well‐being.^22^ The aim of this study was to understand men's experiences of using ambulance services for mental health and/or AOD problems; with a view to identify the positive or helpful aspects of the care they received and the possible areas of care that could be improved. In particular, this article addresses the question: What factors impact on the perceived quality of ambulance care according to men who access ambulance services with mental health and/or AOD problems? This was part of a larger mixed methods study of paramedics' attitudes, confidence and experience in responding to patients with mental health and/or AOD problems.^23^

## 2 METHODS

An exploratory qualitative approach was adopted, which involved semi‐structured interviews. This approach was chosen as an appropriate method to enable the collection of rich and in‐depth data on a sensitive topic.

### 2.1 Participant recruitment

In order to reach a broad population of male patients across Australia, multiple methods of recruitment were implemented. These included advertisements through national mental health organizations e‐newsletters and social media pages, advertisements in online national classifieds (Gumtree and Craigslist), a national media release about the project, posters on community and health service noticeboards, and letters mailed out by some ambulance services inviting males who had accessed the service in the past 12 months to participate in the study. Across all recruitment methods, interested individuals were invited to make contact with the researcher by telephone. Once contact was made, the researcher emailed participants the participant information and consent from, verbally described the project, responded to any questions participants had, and then assessed participant eligibility. Inclusion criteria were as follows: male, aged 18 years or older; had utilized an ambulance service for mental health and/or AOD problems within the past 12 months, and at least 1 month had elapsed after the date of contact with an ambulance service. To ensure that participants were feeling well enough to complete an interview, each individual was screened using the Kessler Psychological Distress Scale (K10). Men that reported a high level of distress (as indicated by a score over 30) were not eligible to participate in the interview and were invited to rescreen at a later date. Verbal consent was gained over the telephone for those participants who were eligible to participate.

Fifty‐seven men expressed interest in participating in the study; of these, 49 met the inclusion criteria, and 30 consented to participate. Reasons eligible individuals declined to participate included; they were no longer interested in participating, no longer had time to complete the interview, or wanted to be compensated at a higher rate for their participation.

### 2.2 Data collection

Individual qualitative interviews were conducted by NF, MS and KE between August 2016 and March 2017. Authors involved in conducting interviews were not employed by an ambulance service and had no prior relationship or interaction with participants. A semi‐structured interview guide was used, which was developed from a review of literature and discussion amongst the authors. Interviews explored participants' reasons for using ambulance services, views about the quality of care received, and the types of services or initiatives that might be helpful for seeking help for mental health‐ and/or AOD‐related problems. However, this paper focusses on participant's views of the quality of care received, as this area of enquiry stimulated considerable discussion and elicited particularly rich responses. The duration of each interview was 50 minutes on average (ranging from 30 to 73 minutes). All interviews were conducted by telephone and were recorded. The study was approved by five human research ethics committees in different states and territories in Australia (Eastern Health Human Research Ethics Committee, Monash University Human Research Ethics Committee,  South Eastern Sydney Local Health District Human Research Ethics Committee, South Australia Department for Health and Wellbeing Human Research Ethics Committee, Flinders University Social and Behavioural Research Ethics Committee). Participants were reimbursed with a $30 supermarket voucher for their time and inconvenience.

### 2.3 Data analysis

Transcribed interviews were analysed drawing on principles of the Framework approach to thematic analysis.^24^ The Framework approach was considered particularly appropriate given that it was developed for applied and practice relevant research, and allows themes to emerge inductively from the data as well as to be deductively derived from the relevant literature and study aims.^24^

All coding was completed by the first author. Following transcription of interviews, the framework approach involved initial familiarization with the interview transcripts. NF and KE then coded several transcripts in NVivo (2011), which were used to inform the development of a coding framework. Codes were grouped into categories, which were discussed with MS and modified until consensus was achieved, and a working thematic framework emerged. This framework was then applied to the remaining transcripts by NF, with new codes discussed, added and agreed upon by NF and MS as they arose. Data were then charted to examine the range of experiences and meanings within themes, and relationships between themes. Finally, data were interpreted in the light of our central focus on the factors that contribute to positive and negative experiences of care. In addition to analyst triangulation where authors discussed and agreed upon codes, other strategies were employed to enhance rigour including the incorporation of diverse experiences as well as those of participants with different mental health and/or AOD concerns.^25^ We also employed reflexive techniques, such as meeting to discuss how ideas of quality care were filtered through the interpretive lens of the researchers.^25^ This was important in deepening our analysis and obtaining consensus around the interpretation of participant accounts, as some of the authors worked in ambulance services whilst others did not.

## 3 RESULTS

### 3.1 Participant characteristics

In total, 30 males who had accessed an ambulance service for a mental health and/or AOD problem between one and 12 months ago participated in the study. The mean age of participants was 40 years. Twenty‐two (72%) were born in Australia, of which 5 (16%) identified as Aboriginal and/or Torres Strait Islander. Just over one‐half of participants (n = 16) were unemployed at the time of the interview, whilst over one‐third (n = 11) were engaged in studies. The majority of participants identified as heterosexual (n = 23, 76%) and most were single at the time of the interview. Most participants (n = 20, 66%) resided in metropolitan locations and lived in either Victoria (n = 11, 36%) or New South Wales (n = 8, 26%); however, there was representation across all Australian states and territories.

Participants accessed an ambulance service for either a mental health concern (n = 15), AOD concern (n = 5) or a combination of mental health and AOD concerns (n = 7) with a minimum of 1 month and less than 12 months, post the time of contact. Some (n = 8) also presented with either self‐harm, suicidal ideation or attempt in addition to other mental health or AOD concerns, or exclusively for suicidal ideation/attempt (n = 3), without other mental health and/or AOD concerns. Whilst participants often had multiple concerns, the most common specific concerns reported included anxiety or panic attack (n = 15), depression (n = 13), intoxication (n = 9), overdose (n = 3) and psychosis or hallucinations (n = 3). Those who reported AOD use were often intoxicated on more than one substance, with alcohol (n = 8) and methamphetamine (n = 2) being the most commonly reported (Table 1).

**Table 1:** Participant characteristics

Participant characteristics (n = 30)		
Age	Mean	Range
	40	21‐67

	n	%
Presenting issue		
Mental health^a^	15	50.0
AOD	5	16.7
Mental health & AOD^b^	7	23.3
Suicidal ideation/attempt^c^	3	10.0
Cultural background/place of birth		
Aboriginal & Torres Strait Islander (ATSI)	5	16.7
Born in Australia but not ATSI	17	56.7
Born outside of Australia	8	26.7
Sexual orientation		
Heterosexual	23	76.7
Other	7	23.3
Marital status		
Single	22	73.3
Currently married/defacto	4	13.3
Divorced/separated	4	13.3
Highest professional education qualification		
Year 10 high school	6	20.0
Year 12 high school	7	23.3
Technical or vocational college	7	23.3
University	10	33.3
Employment status		
Full‐time	7	23.3
Part‐time	2	6.7
Casual	5	16.7
Unemployed	16	53.3
Undertaking studies		
Full‐time	6	20.0
Part‐time	5	16.7
Not undertaking studies	19	63.3
Location		
Metro	20	66.7
Regional	6	20.0
Rural	4	13.3

a Also includes self‐harm, suicidal ideation/attempt.

b Also includes self‐harm, suicidal ideation/attempt.

c Exclusively suicidal ideation/attempt.

### 3.2 Themes

Three main and interconnected themes were abstracted from the data relating to participants' experiences of care: professionalism and compassion, communication and handover. The themes contributed to positive or negative experiences, depending on how they emerged in participants' accounts. The first theme, *professionalism and compassion,* referred to participants' perceived competence of paramedics and the care provided, often referencing displays of compassion, or conversely a lack of compassion. The second theme, *communication,* referred to how participants were spoken to (or not spoken to), the language used and the types of comments that were directed towards participants. The third theme, *handover,* included participants' experiences of being transferred to emergency department staff, in which communication was also influential. The two participants who presented with methamphetamine issues consistently reported negative experiences in each of these three themes.

### 3.3 Professionalism and compassion

Most participants used the word “professional” when describing their positive experiences with paramedics. Professionalism was often reflected in a combination of other positive attributes that are associated with exceptional health care, such as empathy and compassion, where participants described paramedics as being caring or understanding and having a calming effect on them. Conversely, participants' accounts of negative care experiences often lacked professionalism, and in these instances, they commented on the absence of care and comfort in their interactions with paramedics.

### 3.4 Presence of professionalism and compassion

Professionalism and compassion were central to positive experiences of care recounted by most participants:

I've called them up at every “retarded” (very late) time of night. I've experienced them to be very professional, very caring. Initially, I didn’t call them a lot of times because I was worried about that… I was worried if I don’t have a broken leg they might not take it seriously.(Interview 33, aged 27 years, mental health)

This quote highlights a common concern that a mental health issue (including a mental health crisis) could be viewed as a less legitimate reason to engage ambulance services, which might be an access barrier for people who are seeking help for this type of issue. However, as the quote illustrates, the professionalism and care shown by paramedics could act to dispel the perception that mental health issues constituted an “illegitimate” reason to contact ambulance services.

Descriptions of paramedics being empathetic, polite and caring were also mentioned frequently in positive experiences with ambulance services. One participant described being initially hesitant to accept paramedics help:

I didn't let them in at first but I spoke to them through the door for a while. I eventually let them in. They were very understanding and caring, which was great, and sympathetic. So yeah, it was just a matter of getting comfortable.(Interview 4, age 45, mental health and AOD/self‐harm/suicide attempt)

Although the participant did not initially allow paramedics to enter his home, paramedics were able to persist, gain trust through displays of empathy and care, and were eventually granted access to provide care. This account illustrates how persistent displays of empathy can shift tense situations, enable people to feel comfortable and ultimately result in positive outcomes and experiences for patients. Similarly, other participants described feelings of comfort and feeling reassured by paramedics' ability to provide appropriate care, which often had a calming effect:

They knew what to expect and they kept me calm. That was all that was needed. They understood that I was beside myself (distraught) and they took me to the hospital. I spoke to mental health [professionals] and I got some medication, and I started feeling well again.(Interview 32, aged 44, AOD and mental health)

### 3.5 Lack of professionalism and compassion

Whilst most participants experienced positive interactions, some described experiences in which they did not receive the compassion they desired. For example, one participant described wanting:

Just a little bit more care and understanding and effort when it comes to mental health. But the effort and the response that goes into the (patients) callers…that had a stroke or a heart attack then people will be “climbing all over you” (very responsive). But for a mental health issue or a self‐harm issue, or something like that, on occasion I waited over an hour for an ambulance, so it's quite different.(Interview 16, age 26, AOD and mental health/self‐harm)

Here, the participant expresses that he did not feel his mental health issue was given a high priority. Furthermore, as someone with a mental health issue, he, like others, felt he was being judged as less worthy of the same level of medical care that would be given to patients with non‐mental health‐ and/or AOD‐related medical emergencies. In part, this experience may be impacted by the broader system response; however, the participant relates a delay in service response to judgemental attitudes of ambulance staff. A participant with a crystal methamphetamine‐related presentation also provided a similar example of feeling judged whilst receiving care:

It was just they couldn't wait to get me in the van just to jab me, to shut me up, because, obviously, there are more important things. There could be someone having a heart attack or someone that needs the ambulances more than I did, because, like, that was self‐inflicted. I'd done that to myself… so I got the feeling that when it comes to ice (crystal methamphetamine) they don't really have much tolerance or much care for you.(Interview 6, aged 35, AOD)

Both participants who presented with methamphetamine‐related issues reported a lack of empathy and understanding towards their situation. After being discharged from hospital with little medical treatment, one participant returned to the hospital on foot a few hours later seeking medical treatment in the emergency department for the same issues. He reflected on his experience of seeking care, saying:

They [paramedics] are not really trained for certain situations in Australia and apparently especially drug abuse… young or even later career ambulances paramedics, they are not experienced enough to deal with that.(Interview 11, aged 40, AOD)

Overall, these exemplars illustrate how compassion and professionalism, or lack of, can shape a patient's experience of paramedic care, leading to a positive experience when greater levels of professionalism and compassion are shown, or an unfavourable experience when these qualities are lacking.

### 3.6 Communication and attitudes

One of the main ways in which care and compassion was shown was through paramedic communication. Communication varied and affected participants' experience of care greatly. Participants who were communicated to clearly, had processes explained, and felt their concerns were listened to, had positive experiences of care, whilst those who experienced poor communication or judgemental comments reported unfavourable experiences of care.

### 3.7 Good communication and non‐judgemental attitudes

Participants reporting experiences of positive communication often described communication that was non‐judgemental, and egalitarian, which made them feel reassured and cared for. For example, one participant explained:

Yeah, look they asked for permission to examine me. They were extremely professional; they asked me how I'd gotten into this condition. Very non‐judgemental about the whole thing; … they work in a profession when they never know when things are going to go “south” (wrong). So they were very cautious and very verbally gentle with me. [They] let me run the decision‐making process as much as they could allow.(Interview 14, aged 55, AOD and mental health)

This illustrates an excellent example of positive communication and patient‐centred care, where the participant describes how he felt listened to and respected, whilst also feeling that he had a level of autonomy to participate in the decision‐making process about his care.

Other participants also described the value of personalized and humanizing style of communication:

We started talking about different parts of the town that we loved and we were talking about members of the community that we'd both known. Things that were a little bit more personal that put things back in the context of humanity in a realistic sense. It wasn't clinical. It wasn't too excessive. They just… focussed on having just the human interaction, and that was exactly what I needed at that time to sort of overcome it.(Interview 20, aged 24, mental health/suicidal ideation)

This quote underscores the potential positive effects of good communication, which reinforces the idea that good communication, can also be viewed as a therapeutic technique to help treat patients. Similarly, another participant recalled how a paramedic was able de‐escalate a situation between police and himself:

He [the paramedic] knew what he was doing. He knew that he could quickly assess the situation and he calmed everything down. He also calmed the police down and he acted very much as intermediary just going (saying), “just leave this guy alone. If you just leave him alone he'll come quite happily.” I don't know whether that's his job but he just saw the situation out (calmed things down) because I was… getting really angry with them bursting into my house and it's 10 o'clock in the morning. So, he did an excellent job so I couldn't fault him at all.(Interview 30, aged 41, mental health)

For other participants, the calming effect of communication came less from the content of what was being said but from the sound or tone of a paramedic's voice:

I don't know if it was because there were two ladies that attended, that even their voice control and the tone of their voice was soothing.(Interview 1, aged 67, mental health)

Whilst this quote illustrates that, the tone or sound of a voice could be calming, these qualities were not mentioned in relation to male paramedics. Thus, this participant's account may reflect gendered notions of women as nurturing and comforting.

### 3.8 Poor communication and judgemental attitudes

Whilst there were participants who had positive experiences of paramedic communication, some reported experiences of poor communication. In these instances, communication was not used to de‐escalate situations, or was replaced with a focus on restraint and/or sedation as a management tool with little explanation to the participant.

One participant explained that he found the experience of chemical sedation confronting and thought that:

The police and ambulance lack the therapeutic tools to diffuse the situation without conflict and sedation.(Interview 16, aged 26, AOD and mental health/self‐harm)

Another participant also pointed to a lack of communication in regards to the administration of sedation:

Tell me what you're giving me for starters, so I know. Obviously it’s a sedative, but at least give me the name (of the drug) so I know what it is; talk to me a little bit more, ask me questions like, "why, how come that it led up to that particularly at 2:30 in the morning,” and, yeah, maybe just a little bit more conversation in the van (ambulance) before knocking you out (sedating me), I suppose.(Interview 6, aged 35, AOD and mental health)

Some participants also discussed negative experiences of communication beyond restraint/sedation, such as judgemental comments:

Some of them [paramedics] are quite, well, judgemental, I suppose. One said to me, “we could be out saving someone's life rather than transporting you to hospital for a check‐up.” I thought, “well, that’s rather rude, I didn’t ask you to come get me.”(Interview 15, aged 51, mental health/suicidal ideation)

These comments about participants' worthiness of care (or lack of) may reinforce a perception that mental health and/or AOD concerns are not legitimate reasons for contacting an ambulance, as described earlier. Similarly, after regaining consciousness from a heroin overdose, one participant recalled that:

I had one paramedic say: “you're too old to be doing this,” and that was it. I basically had no communication. I respect what they're doing and she was quite right. I was quite embarrassed.(Interview 36, aged 57, AOD)

As illustrated in the above account, this form of judgemental communication can lead to feelings of shame or embarrassment for men with mental health and/or AOD concerns.

Silence and lack of communication were also evident as no participants mentioned receiving any information or referrals (around mental health and/or AOD) in their interactions with paramedics as part of their care.

### 3.9 Handover to emergency department clinicians

For particpants who were transported, their ambulance care journey was completed when handover to emergency department clinicians occurred. Although this is the final interaction between patients and paramedics, it emerged as an important component in participants' experiences of care and had the potential to be a positive or negative experience depending on the level and quality of communication at handover.

### 3.10 Positive handover interactions

For some participants, handover by paramedics involved a smooth transition to emergency department and hospital care. Participants giving examples of positive handover experiences described the handover process being fast and efficient and also feeling well informed by attending paramedics and/or hospital staff.

It was pretty quick. I got in straight away…They [paramedics and hospital staff] communicated with each other what had happened… I can remember them briefing each other… They knew what happened, but they were asking me how I was feeling.(Interview 48, aged 30, AOD)

In this exemplar, the participant describes a positive experience that is timely and demonstrates an efficient handover process with clear communication between paramedics and hospital staff, which also shows care for the participant. Another participant also described a positive handover experience:

I found it was really good because they actually explained it [the handover process] even though…the alcohol was kicking in and…I couldn't hold a straight [conversation]. Yeah, but they managed to actually, yeah, managed to tell the story and stuff.(Interview 47, aged 32, mental health and AOD/suicidal ideation)

In this account, the participant benefited from good explanation and communication during handover, despite the challenging circumstances of intoxication.

### 3.11 Negative handover interactions

However, there were other examples where participants had negative handover experiences characterized by poor communication and a sense of feeling isolated and peripheral to the care provided. For instance, one participant explained:

That they're [paramedics and hospital staff] talking as if I'm not there, but clearly they know I am, because it's about you and all that sort of stuff. It just makes you feel like an idiot or a child or stupid, really.(Interview 7, aged 39, AOD and mental health/suicide attempt/self‐harm)

In this quote, it is evident that poor communication during handover can make a patient feel juvenile or “stupid.” For another participant, the handover between the paramedic and hospital staff was a difficult experience, forcing the participant to repeat his story:

I was in quite a vulnerable situation and I'm not sure how much conversation was had between the paramedics and the hospital that I was going to. As soon as I got to hospital, I had to pretty much explain why I was there. As if, like the first time wasn't hard enough. I felt it was quite unprofessional. I don’t know whose fault that was but I think that just bridging that gap a little bit more in that transition would be good, to make sure that the people that you're being handed over to know your situation.(Interview 20, aged 24, mental health/suicidal ideation)

This exemplar illustrates that when the responsibility for communicating during handover is left solely to patients, it is not only onerous; but can also be traumatizing to have to re‐explain a situation after experiencing a mental health crisis.

## 4 DISCUSSION

This study provides a rare exploration of men's experiences of using ambulance services for mental health and/or AOD problems and illustrated three interconnected factors that influenced their care in different ways: professionalism and compassion, communication and handover to emergency department clinicians. As we will discuss, the findings of this study also give rise to a number of implications in terms of improving the quality of ambulance care for people with mental health and/or AOD issues.

In the first theme, professionalism and compassion, men who accessed ambulance services for mental health and/or AOD concerns greatly valued paramedics who displayed such attributes, and conversely were adversely affected when these attributes were not present in interactions. This finding is consistent with those from a qualitative study conducted in England, in which participants described how the perceived professionalism of health professionals reduced anxiety levels in patients and created confidence in the quality of care provided by the ambulance service.^21^ Although Togher et al's^21^ study was inclusive of perspectives from a much broader range of presentations types (including mental health), a similar set of interpersonal skill attributes that patients described as “professional” were identified. These included being “calm, kind and informative,” which were equally valued regardless of presentation type.^21^ ^(p2956)^ Similarly and consistent with the findings of our study, results from a qualitative study of patient reports of being “badly treated” by ambulance service found that patients suffered when exposed to judgemental attitudes or when interactions with paramedics were perceived as lacking empathy or compassion.^26^ As noted, this may be the experience for patients who present with methamphetamine issues, which could be related to recent stigmatizing portrayals of people who use methamphetamines via the media, polices and public health campaigns.^27^, ^28^

Given the stigma associated with mental health and AOD issues, experiences of professionalism and compassion by health professionals, such as paramedics, are likely to be highly valued. As our findings suggest, the absence of professionalism and compassion may not only result in negative experiences of care but also perpetuate self‐stigma, which may act as a disincentive to further health and social service utilization.^15^, ^29^ This could result in serious adverse consequences, including death, particularly in the case of self‐harm, overdose or mental health crises.

In the second theme, communication emerged as a critical way of influencing patients' experiences of care. Consistent with the findings of other studies,^21^, ^30^ good communication underpins positive experiences of care by making people feel they are being listened to and cared for in an appropriate and patient‐centred way. In terms of what constitutes good communication, participants highlighted the importance of paramedics' listening to them, being non‐judgemental, sensitive and encouraging them to be active participants in the interaction. Similarly, Togher et al^21^ found that feeling listened to and receiving a clear explanation from paramedics made patients feel at ease and reassured. They also found that paramedic communication style, including content and manner, was important for patients, which is also reflected in some participants' experiences in our study. For example, negative experiences often contained communication content by paramedics that was judgemental and reinforced self‐stigma, whilst some patients who had positive experiences commented not only on the communication content but also on the soothing manner in which paramedics communicated with them.

A lack of communication could also be potentially harmful to patient outcomes in other ways, particularly in the case of patient referral and information provision by paramedics. Participants did not report receiving linkage to ongoing care or information on how to prevent or manage their condition. This absence of health promotion practices is noted in other studies, where it is not part of paramedic routine practice, with paramedic education and practice guidelines traditionally focussing on treatment of acute illness rather than public health interventions.^31^ This may mean people experience delays in receiving appropriate care and treatment in the community, during which time the chronicity and severity of their problems may escalate, which in turn, may affect treatment and recovery outcomes adversely.

In the third theme, participants' experiences of the handover to emergency department clinicians featured prominently in participants' accounts of the quality of care they received. As our research indicates, handover can be a time when communication breakdown occurs and patients are left with minimal care and/or needing to repeat their story. Whilst research has indicated that professional's expectations and experiences often are likely to influence quality of handover, results have also indicated that communication, in particular a lack of active listening, has been an issue in handovers.^32^ In a qualitative study analysing patients' experiences of handover, communication and bedside manner were also found to impact on patient satisfaction levels during the handover experience, and that patients desired improved communication during their hospital handovers.^33^ However, consistent with other studies,^21^ these issues can be averted and continuity of care can be assured if paramedics clearly communicate a client's situation and story to emergency staff as well as updating the patient about what is and will be happening.

### 4.1 Strengths and limitations

This study was conducted in the context of ambulance care provided to men with mental health and/or AOD issues across Australia. Whilst qualitative research is generally not concerned with generalizability, the discussion of our findings in the context of other research illustrates that the findings may be applicable more broadly. However, participants in this study self‐selected which may have affected results. For example, participants may have had a greater propensity to discuss mental health and/or AOD issues than other men who had accessed ambulance services. Furthermore, a K10 screening criteria were applied, which excluded individuals who were experiencing high levels of distress from participating. Hence, our interviews only captured those who were classified at the less severe end of the depression and anxiety scale at the time of interviewing. Grouping mental health and AOD presentations together as the focus of the study may have obscured the unique experiences of patients with particular issues. Despite this, our analysis was sensitive to diverse experiences (positive and negative) of care, including the particularly negative experiences of patients who used methamphetamines. However, it should be noted that our sample included only two participants with methamphetamine issues. Thus, future research is needed to explore the care experiences of a larger sample of patients who use methamphetamines.

### 4.2 Implications

Overall, our findings reiterate broader calls for improving the quality of ambulance care for people presenting with mental health and/or AOD issues.^11^, ^12^ A range of measures could be included to achieve this, including an educational approach to address potential skills gaps around communication. This could include training around therapeutic communication skills in pre‐qualification university curricula and in‐service education programs. Low‐cost strategies, such as the provision of referral and information cards, could be piloted in order to facilitate access to appropriate support and ongoing care for patients who need/desire it. Paramedics who demonstrate good communication skills and empathic attitudes could also act as “mental health champions” within ambulance services in order to help cultivate an organizational culture in which stigmatizing attitudes are considered unacceptable. Stigma reduction campaigns and initiatives could also be useful in facilitating empathy and high‐quality care.^34^ Consideration should also be given to reviewing policies and clinical practice guidelines to ensure they provide a framework that reflects a broader view of practice, beyond the current medically dominated scope of practice.^23^ Future research is needed to examine the impacts and experiences of initatives for improving the quality of ambulance care for people with mental health and/or AOD issues.

## CONFLICT OF INTEREST

The authors have declared that they have no competing interests. In the past three years, DL has received speaking honoraria from AstraZeneca, Indivior, Janssen, Servier and Shire, and has provided consultancy advice to Lundbeck and Indivior. In the past three years, TMcC and DL were recipients of an educational grant from Janssen‐Cilag to assist with writing a book for family caregivers of people with schizophrenia.

## References

[1] Australian Bureau of Statistics . National Health Survey: First Results, 2014‐15. Published December 8, 2015. Accessed October 31, 2018.

[2] Australian Bureau of Statistics . Causes of Death, Australia, 2017. Published September, 2018. Accessed October 31, 2018.

[3] Teesson M , Hall W , Lynskey M , Degenhardt L . Alcohol‐ and drug‐use disorders in Australia: implications of the National Survey of Mental Health and Wellbeing. Aust N Z J Psychiatry. 2000;34(2):206‐213. 10789525 10.1080/j.1440-1614.2000.00715.x

[4] Harrison C , Britt H . General practice – workforce gaps now and in 2020. Aust Fam Physician. 2011;40(1–2):12‐15. 21301686

[5] Seidler ZE , Dawes AJ , Rice SM , Oliffe JL , Dhillon HM . The role of masculinity in men’s help‐seeking for depression: a systematic review. Clin Psychol Rev. 2016;49:106‐118. 27664823 10.1016/j.cpr.2016.09.002

[6] Clement S , Schauman O , Graham T , et al. What is the impact of mental health‐related stigma on help‐seeking? A systematic review of quantitative and qualitative studies. Psychol Med. 2015;45(1):11‐27. 24569086 10.1017/S0033291714000129

[7] Yousaf O , Grunfeld EA , Hunter MS . A systematic review of the factors associated with delays in medical and psychological help‐seeking among men. Health Psychol Rev. 2015;9(2):264‐276. 26209212 10.1080/17437199.2013.840954

[8] Galdas PM , Cheater F , Marshall P . Men and health help‐seeking behaviour: literature review. J Adv Nurs. 2005;49(6):616‐623. 15737222 10.1111/j.1365-2648.2004.03331.x

[9] Productivity Commission . Ambulance Services – Report on Government Services 2018. Published January 25, 2018. Accessed, October 16, 2019.

[10] Roberts L , Henderson J . Paramedic perceptions of their role, education, training and working relationships when attending cases of mental illness. J Emerg Prim Health Care. 2009;7(3):1.

[11] Prener C , Lincoln AK . Emergency medical services and “psych calls”: examining the work of urban EMS providers. Am J Orthopsychiatry. 2015;85(6):612. 26192707 10.1037/ort0000077

[12] Roggenkamp R , Andrew E , Nehme Z , Cox S , Smith K . Descriptive analysis of mental health‐related presentations to emergency medical services. Prehosp Emerg Care. 2018;22(4):399‐405. 29364746 10.1080/10903127.2017.1399181

[13] Lloyd B , Gao CX , Heilbronn C , Lubman DI . Self‐Harm and Mental Health‐Related Ambulance Attendances in Australia: 2013 Data. Fitzroy, Victoria: Turning Point; 2015.

[14] Lloyd C . The stigmatization of problem drug users: a narrative literature review. Drug‐Educ Prev Polic. 2013;20(2):85‐95.

[15] Reavley NJ , Jorm AF . Stigmatizing attitudes towards people with mental disorders: findings from an Australian National Survey of Mental Health Literacy and Stigma. Aust N Z J Psychiatry. 2011;45(12):1086‐1093. 22023236 10.3109/00048674.2011.621061

[16] Reavley NJ , Mackinnon AJ , Morgan AJ , Jorm AF . Stigmatising attitudes towards people with mental disorders: a comparison of Australian health professionals with the general community. Aust N Z J Psychiatry. 2014;48(5):433‐441. 23943633 10.1177/0004867413500351

[17] Rees N , Rapport F , Thomas G , John A , Snooks H . Review: perceptions of paramedic and emergency care workers of those who self harm: a systematic review of the quantitative literature. J Psychosom Res. 2014;77:449‐456. 25263398 10.1016/j.jpsychores.2014.09.006

[18] Phillips LA , Shaw A . Substance use more stigmatized than smoking and obesity. J Subst Use. 2013;18(4):247‐253.

[19] Bogomolova S , Tan PJ , Dunn SP , Bizjak‐Mikic M . Understanding the factors that influence patient satisfaction with ambulance services. Health Mark Q. 2016;33(2):163‐180. 27295008 10.1080/07359683.2016.1166864

[20] Gill L , White L . A critical review of patient satisfaction. Leadersh Health Serv. 2009;22(1):8‐19.

[21] Togher FJ , O'Cathain A , Phung V‐H , Turner J , Siriwardena AN . Reassurance as a key outcome valued by emergency ambulance service users: a qualitative interview study. Health Expect. 2015;18(6):2951‐2961. 25303062 10.1111/hex.12279 PMC5810705

[22] Kinsler JJ , Wong MD , Sayles JN , Davis C , Cunningham WE . The effect of perceived stigma from a health care provider on access to care among a low‐income HIV‐positive population. AIDS Patient Care STDS. 2007;21(8):584‐592. 17711383 10.1089/apc.2006.0202

[23] McCann TV , Savic M , Ferguson N , et al. Paramedics’ perceptions of their scope of practice in caring for patients with non-medical emergency-related mental health and/or alcohol and other drug problems: A qualitative study. PLoS ONE. 2018; 13(12): e0208391 10.1371/journal.pone.0208391 30543663 PMC6292637

[24] Ritchie J , Spencer L . Analysing qualitative data In: Bryman A , Burgess B eds. Qualitative Data Analysis for Applied Policy Research. London, UK: Routledge; 1994:173‐194.

[25] Mays N , Pope C . Assessing quality in qualitative research. Brit Med J. 2000;320(7226):50‐52. 10617534 10.1136/bmj.320.7226.50 PMC1117321

[26] Ahlenius M , Lindström V , Vicente V . Patients’ experience of being badly treated in the ambulance service: a qualitative study of deviation reports in Sweden. Int Emerg Nurs. 2017;30:25‐30. 27567212 10.1016/j.ienj.2016.07.004

[27] Douglass CH , Early EC , Wright C , et al. “Just not all ice users do that”: investigating perceptions and potential harms of Australia's Ice Destroys Lives campaign in two studies. Harm Reduct J. 2017;14:1‐8. 28705259 10.1186/s12954-017-0175-9 PMC5513120

[28] Dwyer R , Moore D . Enacting multiple methamphetamines: the ontological politics of public discourse and consumer accounts of a drug and its effects. Int J Drug Policy. 2013;24(3):203‐211. 23540297 10.1016/j.drugpo.2013.03.003

[29] Room R , Trotter RT II , Robert T , Paglia A , Üstün TB . Cross-cultural views on stigma, valuation, parity and societal values towards disability In: Üstün B , Chatterji S , Bickenbach JE , Trotter RT II , Room R , Rehm J , Saxena S , eds. Disability and Culture: Universalism and Diversity. Seattle, WA: Hogrefe & Huber Publishers; 2001:247‐297.

[30] Attree M . Patients' and relatives' experiences and perspectives of 'good' and 'not so good' quality care. J Adv Nurs. 2001;33(4):456‐466. 11251733 10.1046/j.1365-2648.2001.01689.x

[31] Comans TA , Currin ML , Quinn J , Tippett V , Rogers A , Haines TP . Problems with a great idea: referral by prehospital emergency services to a community‐based falls‐prevention service. Inj Prev. 2013;19:134‐138. 22101100 10.1136/injuryprev-2011-040076

[32] Crilly JL , Keijzers GB , Tippett VC , et al. Expanding emergency department capacity: a multisite study. Aust Health Rev. 2014;38:278‐287. 24869756 10.1071/AH13085

[33] Wray CM , Farnan JM , Arora VM , Meltzer DO . A qualitative analysis of patients' experience with hospitalist service handovers. J Hosp Med. 2016;11(10):675‐681. 27167097 10.1002/jhm.2608

[34] McCann TV , Savic M , Ferguson N , et al. Recognition of, and attitudes towards, people with depression and psychosis with/without alcohol and other drug problems: results from a national survey of Australian paramedics. BMJ Open. 2018;8(12):e023860. 10.1136/bmjopen-2018-023860 PMC6286471 30514822

